# Evaluation of the Relationship Between Job Stress Level, Adherence to the Mediterranean Diet, and Phytochemical Index

**DOI:** 10.3390/nu17152469

**Published:** 2025-07-29

**Authors:** Bengi Çetiner Bingül, Murat Baş

**Affiliations:** 1 Department of Nutrition and Dietetics, Institute of Health Sciences, Acibadem Mehmet Ali Aydınlar University, 34752 Istanbul, Turkey; bengi.cetiner@fbu.edu.tr; 2 Department of Nutrition and Dietetics, Faculty of Health Sciences, Fenerbahçe University, 34758 Istanbul, Turkey; 3 Department of Nutrition and Dietetics, Faculty of Health Sciences, Acibadem Mehmet Ali Aydınlar University, 34752 Istanbul, Turkey

**Keywords:** job stress, nutrition, Mediterranean diet, anthropometric measurements, phytochemical index

## Abstract

Background/Objectives: Job stress negatively affects physical and psychological health and can lead to behavioral changes such as unhealthy eating. This study aimed to evaluate the relationship between job stress levels, adherence to the Mediterranean diet, and the phytochemical index (PI). Methods: The study included 200 healthy individuals aged 18–50 working at the Tuzla Gum Factory. Data were collected through demographic and dietary questionnaires, two-day 24-h food records, PI values, and anthropometric measurements. Job stress was assessed using the Job Stress Scale, and Mediterranean diet adherence was assessed with the Mediterranean Diet Adherence Questionnaire. Results: Waist and hip circumference, waist/hip ratio, and BMI were significantly higher in individuals with high levels of job stress (*p* < 0.01). Unskilled workers reported higher stress than professionals (*p* < 0.01). Significant differences were found in carbohydrate and fiber intake among males and in energy, protein, carbohydrate, and vitamin A intake among females with varying stress levels (*p* < 0.01). No significant difference in Mediterranean diet adherence was observed between medium and high stress groups. However, women had higher adherence and PI scores than men (*p* < 0.01). Diet adherence was better among managers than service-sales and technical staff (*p* < 0.01). PI scores were higher in medium stress than high stress individuals (*p* < 0.05) and in those with a higher BMI compared to a normal BMI (*p* < 0.01). Conclusions: Job stress influences both anthropometric parameters and dietary habits. Effective stress management may improve adherence to the Mediterranean diet and phytochemical intake. Workplace strategies supporting healthy eating behaviors are recommended.

## 1. Introduction

Job stress is a prevalent and detrimental aspect of modern work environments. When an individual is exposed to these challenging working conditions, the body activates a stress response to maintain optimal functioning. This reaction can become chronic in a stressful workplace environment, leading to multiple problems that can significantly negatively impact one’s health [1]. Job stress can interfere with cognitive processes and affect an individual’s food choices and nutritional behaviors, disrupting their healthy eating patterns [2]. It can lead to the overconsumption of foods high in calories, fat, or sugar, likely contributing to weight gain. Physical inactivity and lack of time to prepare healthy meals can also increase one’s weight [3]. CareerBuilder, a US-based multinational company, conducted an online survey of 3420 full-time employees to investigate the prevalence of weight gain at work caused by physical inactivity and lack of time, and they found that 56% of the employees gained weight [4]. A study examining the relationship between perceived stress levels in adults and healthy eating attitudes and body weight observed that individuals with ideal healthy eating habits had lower stress levels than others, but perceived stress had no direct effect on body mass index (BMI), with no significant relationship between BMI and healthy eating attitudes. However, stressful situations can cause individuals to turn to unhealthy and packaged food products. These changes in eating habits may lead to weight gain in the long term [5]. When the relationship between stress and eating behavior is examined, emotional eating behavior refers not to physiological hunger but to food consumption in response to emotional states such as stress, anxiety, and sadness. This behavior plays an important role in the eating habits of individuals and has a decisive effect on their general wellbeing [6]. Kendirkiran and Batur showed that an increase in workload and work experience results in higher satisfaction among academic staff and that this satisfaction is associated with healthier eating habits [7]. The Mediterranean diet or other healthy diets containing high amounts of olive oil, fish, fruits, vegetables, white meat, oilseeds, legumes, and unprocessed meat are inversely associated with stress [8]. In a study examining the relationship between job stress and eating behavior, the nutritional patterns of individuals experiencing high job stress levels were far from the Mediterranean diet model, and they had higher energy, saturated fat, and sugar intake than those experiencing low job stress levels [9]. A study involving 162 adult women from Mersin province determined that depression scale scores decreased as the level of compliance with the Mediterranean diet increased. Thus, the Mediterranean diet may be negatively related to stress-related depressive symptoms [10]. Phytochemical-rich diets may yield positive effects on mood and cognitive function in stressful situations through the direct activity of antioxidants and the modulation of enzyme expression or hormone activity [11]. Higher fruit and vegetable consumption has been associated with lower perceived stress levels. However, the relationship between fruit and vegetable consumption and different domains of perceived stress is unclear [12]. In another cross-sectional study examining the relationship between stress symptoms and dietary phytochemical intake, the group with higher phytochemical intake showed significantly lower stress levels [13]. Several studies have reported the association between stress levels and phytochemical intake. However, no study has specifically examined the relationship between job-related stress and phytochemical intake levels. This study aimed to evaluate the relationship between job stress levels, adherence to the Mediterranean diet, and PI.

## 2. Methods

### 2.1. Participants of the Study

This study included 200 healthy individuals (82 females and 112 males) aged 18–50 working in various positions at the Tuzla Gum Factory. The Acibadem Healthcare Institutions Medical Research Ethics Committee approved this study (Approval No: 2020-01/17). All study protocols conformed to the Declaration of Helsinki. Informed consent was obtained from all participants before participating in the study. Participants’ anthropometric measurements, including body weight, height, and waist and hip circumference, were measured using appropriate methods. This study was designed as a cross-sectional study and was conducted between March 2020 and January 2021 at the Tuzla Gum Factory. A convenience sampling method was used to recruit participants from a single industrial workplace.

### 2.2. Anthropometric Measurements

Body weight was measured using a digital scale with a sensitivity of 0.1 kg, with the participants wearing light clothing and no shoes. Height was determined using a non-stretchable tape measure in accordance with the Frankfurt plane. BMI was calculated using the weight (kg)/height (m^2^) formula and was evaluated according to the World Health Organization (WHO) classification. A BMI value below 18.5 kg/m^2^ is classified as underweight, 18.5–24.9 kg/m^2^ as normal, 25–29.9 kg/m^2^ as overweight, 30–34.9 kg/m^2^ as moderate obesity, and above 35 kg/m^2^ as severe obesity. While the participant was standing, waist circumference was measured at the midpoint between the lower rib and the crista iliac crest, and hip circumference was measured at the widest part of the hip. With these values, the waist/hip ratio was calculated and evaluated according to the WHO criteria [14].

### 2.3. Data Collection Tools

Data were collected using a questionnaire form that determined the individuals’ demographic characteristics and general nutritional habits. The Job Stress Scale (JSS) tool was also employed to identify their job stress levels, and the Mediterranean Diet Adherence Evaluation Questionnaire (PREDIMED) was used to determine their consumption of foods characterized by the Mediterranean diet. Additionally, this study conducted a face-to-face interview with each participant, using a questionnaire including food consumption records with a 24 h reminder method for 2 days.

#### 2.3.1. JSS

The validity and reliability of JSS, which was used in this study to assess the participants’ job stress level, were established by Aktaş in Türkiye in 1996. Each item on the scale was scored, and the total score was calculated and classified as follows: low stress (<12 points), medium stress (12–30 points), and high stress (≥30 points) [15]. The internal consistency of the scale was evaluated, obtaining a Cronbach’s α coefficient of 0.826. This value shows that the scale provides reliable and consistent results.

#### 2.3.2. Determination of the Food Consumption Status

Two 24 h daily food consumption records were taken to determine the participants’ energy and nutrient intake, one of which coincided with the weekend. The amounts of food consumed were questioned in detail, and daily averages were calculated. These data were analyzed using the Nutrition Information System (BeBİS 7) program; the adequacy of energy and nutrient intakes was evaluated according to the Turkish Dietary Guidelines (TÜBER 2015) criteria [16,17].

#### 2.3.3. Phytochemical Index (PI)

Data on phytochemical intake were obtained from the food consumption record, evaluated according to the BEBİS program, and calculated using the following PI formula developed by McCarty and Mark in 2004: PI = energy from phytochemical-rich foods (kcal/day)/total energy (kcal/day) × 100. Foods evaluated within the scope of PI are fruits, vegetables (except potatoes), legumes, whole grains, nuts, oilseeds, virgin olive oil, vegetable-fruit juices, and beverages such as wine. The obtained PI values were then divided into quarters (Q1–Q4), and comparisons were made between the groups [18].

#### 2.3.4. PREDIMED

The validity and reliability of the PREDIMED questionnaire, which was used in this study to assess participants’ adherence to the Mediterranean diet, were established by Martínez-González et al. (2012) [19] and adapted to Turkish by Pehlivanoğlu et al. (2020) [20]. The Cronbach’s alpha value was 0.76. This 14-item questionnaire evaluates the consumption frequency of foods such as olive oil, vegetables, fruits, red meat, and fish. The total score was interpreted according to the three categories of adherence to the Mediterranean diet. A total score of <5 indicates low adherence to the Mediterranean diet, 6–9 indicates moderate adherence, and ≥10 indicates high adherence [20].

### 2.4. Statistical Analysis

The IBM SPSS version 23.0 package program was used for statistical data analysis. Data on energy and nutrient intake were evaluated using the BEBİS 7 package program. The categorical variables are presented as numbers and percentages, whereas the continuous variables, summarized using descriptive statistics, are expressed as mean and SD values. The relationship between the categorical variables was examined using the Pearson’s chi-square test, while that between different parameters was determined using the Spearman correlation test. The normal distribution control of the variables was examined using the Shapiro–Wilk test. The Shapiro–Wilk test was used to assess the normality of distributions, and Levene’s test was used to assess the homogeneity of variances before applying one-way analysis of variance (ANOVA). For comparing normally distributed data between two groups, an independent sample *t*-test and ANOVA were used. For comparisons involving three or more groups, ANOVA was applied. When significant differences were found in the ANOVA, the Tukey post hoc test was used for pairwise subgroup comparisons. Within the scope of the reliability study, the internal consistency method was used; thus, the Cronbach alpha coefficient values were examined. A *p*-value below 0.05, within the 95% confidence interval, was considered statistically significant.

## 3. Results

### 3.1. Demographic Characteristics

Table 1 lists the participants’ demographic characteristics. In total, 200 individuals participated in the study, 41% of whom were female and 59% were male. Their mean age was 33.6 ± 9.1 years. Regarding job stress levels, 54.5% experienced moderate stress, and 45.5% experienced high stress.

### 3.2. Anthropometric Characteristics

Table 2 summarizes the BMI and anthropometric measurement values of the participants according to their job stress levels. Waist circumference, hip circumference, and waist/hip ratio values showed statistically significant differences among the participants (*p* < 0.01). The BMI values of those with high stress levels were significantly higher than those with moderate stress levels (*p* < 0.01). All comparisons were made between medium and high stress levels within each gender group.

### 3.3. Job Stress and Professions

Table 3 compares the job stress levels by occupational groups. The highest level of job stress was found in individuals working in jobs that do not require qualifications; their stress levels were significantly higher than those of managers and professionals (*p* < 0.01).

### 3.4. Nutritional Status

Table 4 presents the energy and nutrient intakes of the participants according to their job stress levels. The carbohydrate and fiber intakes of male participants with high stress levels were statistically significantly different compared with those with moderate stress levels (*p* < 0.01). Similarly, the energy, protein, carbohydrate, and vitamin A intakes of female participants with high stress levels were significantly higher than those of the moderate stress group (*p* < 0.01).

### 3.5. Mediterranean Diet Compliance Points

Table 5 presents the relationship between the participants’ Mediterranean diet compliance scores and sex, BMI, occupational groups, and job stress levels. The Mediterranean diet compliance scores of individuals in the medium and high stress groups were similar, demonstrating no significant difference (*p* > 0.05). However, women’s compliance scores were significantly higher than men (*p* < 0.01). When evaluated according to occupational groups, the highest compliance score was found in individuals in managerial positions, while the lowest score was found in individuals working in the service and sales departments. The scores of individuals in the managerial position were significantly higher than those in the service-sales and technician groups (*p* < 0.01). In addition, individuals with a BMI below 18.5 kg/m^2^ had the highest compliance with the Mediterranean diet, while those with a BMI of 35 kg/m^2^ or more had the lowest. However, the difference between the groups was statistically significant (*p* > 0.05). Finally, the Mediterranean diet compliance scores of individuals with medium and high stress levels were similar and at a moderate level, showing no significant difference (*p* > 0.05).

### 3.6. PI Scores

Table 6 presents the comparison of PI data according to the demographic characteristics of the participants. The PI scores in the medium stress group were significantly higher than those in the high stress group (*p* < 0.05). Women had significantly higher PI scores than men (*p* < 0.01). The PI score also significantly differed between individuals with normal BMI and those with obesity (*p* < 0.01). In addition, the BMI of individuals increased as their PI values decreased. Individuals working as managers had the highest PI values, whereas those working in the service and sales departments had the lowest. However, the phytochemical intake values did not significantly differ among the occupational groups (*p* > 0.05).

## 4. Discussion

This study demonstrated that individuals with high job stress levels exhibited negative effects on anthropometric measurements, Mediterranean diet compliance scores, and PI values. Additionally, women had higher compliance and index scores than men. These findings reveal the effect of job-related stress on individuals’ nutritional behaviors and health indicators.

### 4.1. Job Stress and Anthropometric Measurements

Recently, stress has been reported to increase appetite and trigger high-energy food consumption, possibly leading to obesity, particularly by causing an increase in the cortisol hormone [21]. In the present study, significant relationships were found between job stress and anthropometric measurements. Particularly, higher BMI, waist circumference, and waist/hip ratio values showed the possible effects of stress on body composition in the high stress group. This finding is in line with the results of de Lira et al. (2022), who observed that higher perceived stress was associated with increased adiposity and waist circumference among adult workers in Brazil [22].

In terms of BMI, the lowest stress level was noted in individuals with a normal body range and the highest in those with obesity. Therefore, high stress levels may be associated with body weight gain. Similarly, Geda et al. reported that, in Canada, the risk of obesity increased significantly as the level of job-related stress increased [23]. However, this previous finding is generally applicable to the general population. In the present study, this relationship was specifically evaluated among individuals working in a specific sector. Of note, the effects of stress on BMI are linked not only to physiological factors but also to psychosocial factors such as individuals’ perception of stress, coping style, and environmental support systems. Regarding occupational groups, individuals working in jobs that do not require qualifications tended to exhibit higher stress levels, while those in managerial and professional positions appeared to have lower stress levels. Thus, factors such as the level of job control and the nature of the tasks may affect stress levels. Positions such as directorships may allow for better stress management due to greater decision-making authority and autonomy. This interpretation is supported by studies showing that white-collar workers, who typically have more influence at work and control over their tasks, report lower levels of work-related stress compared to blue-collar workers [24,25].

However, these relationships cannot be explained solely by the nature of the job; psychosocial variables such as individuals’ perception of stress, personality traits, and coping strategies may also play a role. Therefore, these interactions should be examined with models that include more psychosocial variables in future studies.

### 4.2. Stress and Nutritional Intake

High levels of job stress can negatively affect individuals’ eating habits, resulting in irregular meal consumption, unhealthy food choices, and inadequate intake of certain nutrients. These effects can have a direct impact on an individual’s energy level, cognitive performance, and ability to cope with stress [26]. In the present study, significant differences were observed between some nutrient intakes in individuals with moderate and high stress levels. Men with high stress levels had higher carbohydrate and fiber intakes, while women had notably higher energy, protein, carbohydrate, and vitamin A intakes. These findings align with previous studies showing that, under stress, individuals tend to consume more energy-dense and often more refined foods. For example, Hemiö et al. reported that an increase in work-related stress and night shift frequency was associated with a higher intake of saturated fat and a decrease in micronutrient intake, particularly among men [27].

In a study conducted by Miki et al., Japanese workers with high stress levels had a decreased intake of micronutrients such as calcium, magnesium, iron, and zinc [28]. However, in the current study, the intake of some nutrients increased among women. This difference may be explained by the differences in sex-based metabolic needs, emotional eating tendencies, and stress-coping styles. Interestingly, fiber intake increased with increasing stress in men, suggesting that some individuals are more inclined to making healthier choices to cope with stress. Hence, stress does not create the same eating behavior response in every individual; personal awareness, nutritional consciousness, and workplace conditions can influence such a response. Thus, the relationship between job stress and food intake is multidimensional and cannot be explained solely by an increase in energy or a decrease in a single micronutrient. Therefore, stress types (acute vs. chronic), individual coping strategies, and eating behavior patterns should be evaluated together in future studies.

### 4.3. Evaluation of Mediterranean Diet Adherence

The Mediterranean diet stands out among healthy eating patterns, owing to its effectiveness in preventing chronic diseases such as cardiovascular diseases and obesity [29]. In this study, many participants showed moderate adherence to the Mediterranean diet. The compliance scores were notably higher among women than among men. This finding is consistent with previous studies reporting that women are more likely to have healthy eating habits. When evaluated according to occupational groups, individuals in managerial positions showed the highest level of compliance with the Mediterranean diet, whereas service and sales personnel showed the lowest level. Therefore, factors such as socioeconomic level, education status, workload, and food access, which vary depending on the job position, may affect diet quality. In terms of BMI, the Mediterranean diet compliance scores were highest in individuals with a BMI below 18.5 kg/m^2^ but lowest in those with ≥35 kg/m^2^. However, this difference did not indicate a meaningful distinction. This finding suggests that diet quality is related not only to body weight but also to stress level, individual consciousness, and environmental conditions. No significant differences were found between job stress level and Mediterranean diet compliance. Both individuals with medium and high stress levels showed moderate compliance with the diet. This finding is parallel to Gümüş et al.’s study investigating defense industry employees [30]. However, this reflects the multidimensional nature of the relationship between job stress and diet quality; hence, dietary preferences may be affected by indirect means (lack of time, emotional eating, convenience food preferences, etc.) rather than the direct effect of stress. This observation is also supported by a recent study conducted among healthcare workers in Italy, where individuals with higher levels of work stress—especially those experiencing heavy workloads and low job control—showed lower adherence to the Mediterranean diet [31].

### 4.4. Relationship Between the PI and Stress

Phytochemicals are bioactive compounds with antioxidant and anti-inflammatory properties that are naturally found in plant-derived foods such as fruits, vegetables, whole grains, and legumes. These compounds have positive effects on stress management, mood regulation, and cognitive functions, and diets with a high phytochemical content can reduce psychological stress levels [32,33]. In the present study, a noticeable relationship was observed between job stress levels and PI. Participants in the moderate stress group exhibited notably higher PI scores compared to those in the high stress group. This finding shows that, as stress increases, the consumption of foods such as fruits, vegetables, and whole grains decreases. Additionally, women showed higher PI scores than men. This result is in line with previous studies that reported that women are more likely to consume vegetables and fruits [33]. Similarly, Nagler et al. reported that, among blue-collar workers, the consumption of low-nutrient snacks and fruits and vegetables increased as a result of stress and fatigue [34]. The current study findings also support the fact that the tendency toward consuming healthy foods containing phytochemicals decreases, especially when the job stress level increases. Therefore, individuals may move away from healthy food choices under job stress. These findings are consistent with the Nurses’ Health Study, which demonstrated that the higher long-term intake of flavonoids—a major class of phytochemicals—was inversely associated with perceived stress symptoms in adult women over a 10-year follow-up period [35]. Furthermore, a study by Cortes et al. (2022) [36] among Brazilian workers showed that high perceived stress levels were significantly associated with the increased consumption of ultra-processed foods, which are typically low in phytochemicals. This reinforces the notion that psychological stress can alter dietary behavior in a way that reduces phytochemical-rich food intake [36].

When evaluated in terms of BMI groups, the PI scores were higher in individuals with normal weight but lower in individuals with obesity, suggesting an existing relationship between body weight and the phytonutrient density of the diet. Although no statistically significant differences were observed between occupational groups, the PI values of individuals in managerial positions were the highest, whereas those of individuals working in the service and sales departments were the lowest. This trend suggests that environmental and behavioral factors related to the job position affect the access and consumption of foods, especially vegetables and fruits. To date, studies examining the direct relationship between job stress and PI are limited. Nonetheless, our study offers an original contribution as one of the few studies that evaluate both the effect of stress levels on dietary patterns and its link to diets high in phytochemical density.

## 5. Limitations of the Study

This study has some limitations. Given that the study sample consisted of only 200 individuals recruited from a single factory, the generalizability of the findings to other populations, industries, or workplace settings is limited. In addition, this study has a cross-sectional design, and the relationship of job stress with dietary habits and PI was evaluated only in a certain time; thus, the causal relationship cannot be determined. Furthermore, participants’ dietary data may not fully reflect long-term habits because they are based on only 2 days of 24 h consumption records. In addition, the level of job stress was measured using the JSS, which is based on self-reporting, thereby increasing the subjectivity of the responses. Adherence to the Mediterranean diet was assessed with the PREDIMED questionnaire, but the limited scope of this scale and cultural adaptation differences should be taken into consideration. Finally, the study was conducted in only one workplace setting, thereby limiting the generalizability of the results to larger populations.

## 6. Conclusions

The level of job stress may have negative effects on Mediterranean diet compliance and PI. Unhealthy eating habits were found to be more common in individuals with high stress levels; vegetable and fruit consumption decreased, while fast-food consumption increased. Thus, stress-related factors such as time management difficulties, emotional eating behaviors, and physical inactivity constitute obstacles to healthy food choices. These findings highlight the need for the dissemination of holistic interventions for stress management in workplace environments and the implementation of environmental regulations that encourage healthy eating behaviors. These interventions include offering healthy and balanced menus, sharing informative materials, and organizing training seminars for employees. Additionally, introducing evidence-based nutritional models such as the Mediterranean diet and facilitating access to foods high in fiber and phytochemicals can contribute to protecting both the physical and mental health of employees. This study contributes to the literature, given that it is one of the few studies examining the relationships between job stress, Mediterranean diet compliance, and PI. However, intervention-based, multicenter studies with a larger sample size are required to better understand the causality of these relationships. Further intervention and follow-up studies are also recommended to elucidate the causal relationships between job stress and nutritional behaviors and to thoroughly evaluate the effects of healthy food choices on public health.

## Figures and Tables

**Table 1:** Demographic characteristics of the participants.

Variable		Frequency (*n*)	Percentage (%)
Sex	Male	118	59.0
	Female	82	41.0
Age	<25	8	4.0
	26–35	88	44.0
	36–44	61	30.5
	45–54	43	21.5
Marital status	Married	136	68.0
	Single	60	30.0
	Widow	4	2.0
Education Status	Elementary School	48	24.0
	Middle School	14	7.0
	High School	55	27.5
	University	74	37.0
	Master’s Degree	9	4.5
Number of dependents	1–3	160	80.0
	4–6	38	19.0
	>6	2	1.0
Number of children	0	81	40.5
	1	58	29.0
	≥2	61	30.5
Stress	Medium degree	109	54.5
	High degree	91	45.5
Total		200	100

**Table 2:** BMI and anthropometric measurements according to the job stress level.

Job Stress Scale Value						
	Male			Female		
Anthropometric Measurement Variables	Medium Stress	High Stress	*p*	Medium Stress	High Stress	*p*
	Mean ± SD	Mean ± SD		Mean ± SD	Mean ± SD	
Waist circumference (cm)	88.81 ± 8.5	98.48 ± 8.8	0.000 *	76.30 ± 12.0	90.06 ± 13.5	0.000 *
Hip circumference (cm)	99.47 ± 6.9	105.39 ± 7.8		98.34 ± 9.5	107.89 ± 10.3	
Waist/Hip (cm)	0.89 ± 0.05	0.93 ± 0.05		0.77 ± 0.66	0.83 ± 0.07	
Body mass index (kg/m^2)^	25.31 ± 2.80	29.18 ± 3.27		23.86 ± 4.70	28.58 ± 5.27	

Values are presented as mean ± SD. Independent samples *t*-test was used to compare medium and high stress levels within each gender group. * *p* < 0.01 indicates statistical significance.

**Table 3:** Comparison of job stress levels according to the profession groups.

JSS Value			
Professions	*n*	Mean ± SD	*p*
Manager	14	26.28 ± 3.75	0.003 *
Professional	33	28.00 ± 5.36	
Assistant professional	25	27.72 ± 6.71	
Administrative staff	31	30.03 ± 6.22	
Service and sales staff	18	31.11 ± 7.05	
Artists	19	29.21 ± 6.44	
Machine operator, assembly	3	25.66 ± 2.51	
Unskilled workers	57	32.19 ± 5.61	

JSS: Job Stress Scale. Values are presented as mean ± SD. One-way ANOVA followed by Tukey post hoc test was used to compare differences between occupational groups. * *p* < 0.01 indicates statistical significance.

**Table 4:** Relationship between job stress and nutritional status according to sex.

Nutritional Values	Job Stress	Male		Female	
		Mean ± SD	*p*	Mean ± SD	*p*
Energy (kcal)	Medium Stress	2079.3 ± 224.5	0.142	1632.7 ± 290.4	0.002 **
	High Stress	2140.1 ± 227.2		1783.9 ± 277.9	
Protein (g)	Medium Stress	77.8 ± 12.5	0.530	60.5 ± 12.6	0.003 **
	High Stress	79.4 ± 14.1		67.7 ± 17.1	
Fat (g)	Medium Stress	97.9 ± 21.6	0.924	81.4 ± 18.1	0.538
	High Stress	97.6 ± 18.5		83.6 ± 12.0	
CHO (g)	Medium Stress	215.7 ± 42.1	0.003 **	160.5 ± 43.0	0.001 **
	High Stress	229.8 ± 55.3		185.7 ± 48.2	
Fiber (g)	Medium Stress	21.3 ± 5.1	0.002 **	18.5 ± 5.5	0.270
	High Stress	23.2 ± 7.0		20.0 ± 6.6	
Multi S. Fat (g)	Medium Stress	18 ± 6.3	0.932	15.2 ± 5.5	0.746
	High Stress	17.9 ± 5.7		15.6 ± 4.8	
Cholesterol (mg)	Medium Stress	453.6 ± 193.6	0.560	321.9 ± 103.6	0.768
	High Stress	448.4 ± 151.6		329.8 ± 138.8	
Vit. A (µg)	Medium Stress	1121.5 ± 688.1	0.465	1038.6 ± 484.1	0.001 **
	High Stress	1233.8 ± 963.5		1025 ± 262.8	
Carotene (mg)	Medium Stress	2.7 ± 1.7	0.921	3.4 ± 2.6	0.405
	High Stress	2.8 ± 1.7		3.0 ± 1.6	
Vit. E (mg)	Medium Stress	15.6 ± 6.1	0.359	14.8 ± 6.4	0.762
	High Stress	14.6 ± 5.8		14.4 ± 5.7	
Vit. B1 (mg)	Medium Stress	0.9 ± 0.2	0.557	0.8 ± 0.2	0.578
	High Stress	0.9 ± 0.2		0.8 ± 0.2	
Vit. B2 (mg)	Medium Stress	1.4 ± 0.3	0.429	1.1 ± 0.2	0.258
	High Stress	1.5 ± 0.3		1.2 ± 0.3	
Vit. B6 (mg)	Medium Stress	1.3 ± 0.3	0.334	1.0 ± 0.2	0.344
	High Stress	1.2 ± 0.3		1.1 ± 0.2	
Folate (µg)	Medium Stress	333.5 ± 72.2	0.270	280.1 ± 72	0.148
	High Stress	348.9 ± 79.3		306.9 ± 94.3	
Vit. C (mg)	Medium Stress	92.6 ± 39.3	0.525	96.3 ± 49.1	0.827
	High Stress	87 ± 55.2		94.0 ± 44.3	
Sodium (mg)	Medium Stress	4683.8 ± 3700	0.504	3330.2 ± 970.2	0.002 **
	High Stress	5114 ± 3215		3883.5 ± 1158.2	
Potassium (mg)	Medium Stress	2512.1 ± 378.7	0.521	2080.6 ± 464.2	0.09
	High Stress	2459.6 ± 503.9		2218 ± 481.1	
Calcium (mg)	Medium Stress	822.3 ± 157.4	0.060	698.8 ± 194.5	0.178
	High Stress	886.8 ± 211.5		765.8 ± 252.1	
Magnesium (mg)	Medium Stress	314.5 ± 65.8	0.682	271.0 ± 58.6	0.662
	High Stress	319.4 ± 62.3		276.8 ± 59.8	
Phosphorus (mg)	Medium Stress	1216.3 ± 201.3	0.125	977.6 ± 194	0.121
	High Stress	1281 ± 252.9		1054.4 ± 249.9	
Iron (mg)	Medium Stress	11.2 ± 2.3	0.781	9.5 ± 2.2	0.548
	High Stress	11.3 ± 2.0		9.8 ± 2.6	
Zinc (mg)	Medium Stress	11.7 ± 2.4	0.520	9.4 ± 2.0	0.100
	High Stress	12.0 ± 2.6		10.3 ± 2.5	

Values are presented as mean ± SD. Independent samples *t*-test was used to compare medium and high stress levels within each gender group. ** *p* < 0.01 indicate statistical significance.

**Table 5:** Evaluation of the relationship between Mediterranean diet compliance and sex, profession, BMI, and job stress.

Variable		*n*	Mean ± SD	*p*
Sex	Male	118	5.40 ± 2.80	0.000 *
	Female	82	7.30 ± 2.64	
Professions	Manager	14	7.71 ± 2.42	0.006 *
	Professionals	33	6.09 ± 3.38	
	Technicians	25	4.88 ± 2.55	
	Office workers	31	6.13 ± 2.89	
	Service and sales personnel	18	4.56 ± 2.95	
	Artists	19	6.16 ± 2.31	
	Machine operators	3	8.00 ± 3.46	
	Unqualified work	57	6.88 ± 2.65	
BMI	<18.5 kg/m^2^	13	7.00 ± 2.08	0.278
	18.5–24.9 kg/m^2^	65	5.97 ± 2.75	
	25–29.9 kg/m^2^	72	6.63 ± 2.79	
	30–34.9 kg/m^2^	45	5.62 ± 3.31	
	>35 kg/m^2^	5	5.40 ± 3.78	
Stress Situation	Medium stress	109	6.26 ± 2.65	0.683
	High stress	91	6.09 ± 3.18	

Values are presented as mean ± SD. Independent samples *t*-test was used to compare male and female participants. Normality was assessed using the Shapiro–Wilk test, and homogeneity of variances was tested using Levene’s test. * *p* < 0.05 indicate statistical significance.

**Table 6:** Comparison of PI according to sex, profession, BMI, and job stress level.

Phytochemical Index				
Variable		*n*	Mean ± SD	*p*
Sex	Male	118	21.53 ± 11.8	0.004 **
	Female	82	27.46 ± 15.27	
BMI	≤18.5 kg/m^2^	13	35.61 ± 16.00	0.000 **
	18.5–24.9 kg/m^2^	65	27.62 ± 14.68	
	25–29.9 kg/m^2^	72	22.13 ± 11.79	
	30–34.9 kg/m^2^	45	18.94 ± 11.28	
	≥35 kg/m^2^	5	17.67 ± 10.26	
Profession	Manager	14	28.38 ± 10.9	0.216
	Professionals	33	25.01 ± 13.96	
	Technicians	25	23.94 ± 14.68	
	Office workers	31	27.94 ± 17.12	
	Service and sales personnel	18	17.26 ± 11.01	
	Artists	19	21.62 ± 12.29	
	Machine operators	3	20.82 ± 7.22	
	Unqualified work	57	23.17 ± 12.36	
Stress Situation	Medium stress	109	25.73 ± 13.6	0.045 *
	High stress	91	21.85 ± 13.3	

Values are presented as mean ± SD. One-way ANOVA was used to compare differences between groups. Normality was assessed using the Shapiro–Wilk test, and homogeneity of variances was tested using Levene’s test. * *p* < 0.05 and ** *p* < 0.01 indicate statistical significance.

## Data Availability

The original contributions presented in this study are included in the article. Further inquiries can be directed to the corresponding author.

## Abbreviations

The following abbreviations are used in this manuscript:

BMI	Body mass index
CHO	Carbohydrate
JSS	Job Stress Scale
PI	Phytochemical index
PREDIMED	Mediterranean Diet Adherence Survey
WHO	World Health Organization
